# Non-neutralizing Antibodies May Contribute to Suppression of SIVmac239 Viremia in Indian Rhesus Macaques

**DOI:** 10.3389/fimmu.2021.657424

**Published:** 2021-03-16

**Authors:** Nuria Pedreño-Lopez, Brandon C. Rosen, Walter J. Flores, Matthew J. Gorman, Thomas B. Voigt, Michael J. Ricciardi, Kristin Crosno, Kim L. Weisgrau, Christopher L. Parks, Jeffrey D. Lifson, Galit Alter, Eva G. Rakasz, Diogo M. Magnani, Mauricio A. Martins, David I. Watkins

**Affiliations:** ^1^Department of Pathology, George Washington University, Washington, DC, United States; ^2^Department of Pathology, University of Miami Leonard M. Miller School of Medicine, Miami, FL, United States; ^3^Medical Scientist Training Program, University of Miami Leonard M. Miller School of Medicine, Miami, FL, United States; ^4^Nonhuman Primate Reagent Resource, MassBiologics of the University of Massachusetts Medical School, Boston, MA, United States; ^5^Ragon Institute of Massachusetts General Hospital (MGH), Massachusetts Institute of Technology (MIT), and Harvard University, Cambridge, MA, United States; ^6^Wisconsin National Primate Research Center, University of Wisconsin-Madison, Madison, WI, United States; ^7^International AIDS Vaccine Initiative, AIDS Vaccine Design and Development Laboratory, Brooklyn, NY, United States; ^8^AIDS and Cancer Virus Program, Leidos Biomedical Research Inc., Frederick National Laboratory for Cancer Research, Frederick, MD, United States; ^9^Department of Immunology and Microbiology, Scripps Research, Jupiter, FL, United States

**Keywords:** non-neutralizing antibodies, SIV, rhesus macaques (*Macaca mulatta*), immunoadsorption, anti-FcRn Ab, adoptive transfer, antibody depletion

## Abstract

The antiviral properties of broadly neutralizing antibodies against HIV are well-documented but no vaccine is currently able to elicit protective titers of these responses in primates. While current vaccine modalities can readily induce non-neutralizing antibodies against simian immunodeficiency virus (SIV) and HIV, the ability of these responses to restrict lentivirus transmission and replication remains controversial. Here, we investigated the antiviral properties of non-neutralizing antibodies in a group of Indian rhesus macaques (RMs) that were vaccinated with *vif*, *rev, tat, nef*, and *env*, as part of a previous study conducted by our group. These animals manifested rapid and durable control of viral replication to below detection limits shortly after SIVmac239 infection. Although these animals had no serological neutralizing activity against SIVmac239 prior to infection, their pre-challenge titers of Env-binding antibodies correlated with control of viral replication. To assess the contribution of anti-Env humoral immune responses to virologic control in two of these animals, we transiently depleted their circulating antibodies via extracorporeal plasma immunoadsorption and inhibition of IgG recycling through antibody-mediated blockade of the neonatal Fc receptor. These procedures reduced Ig serum concentrations by up to 80% and temporarily induced SIVmac239 replication in these animals. Next, we transferred purified total Ig from the rapid controllers into six vaccinated RMs one day before intrarectal challenge with SIVmac239. Although recipients of the hyperimmune anti-SIV Ig fraction were not protected from infection, their peak and chronic phase viral loads were significantly lower than those in concurrent unvaccinated control animals. Together, our results suggest that non-neutralizing Abs may play a role in the suppression of SIVmac239 viremia.

## Introduction

Antibodies (Abs) are one of the two major components of the adaptive immune response and are key in vaccine development. Indeed, vaccine efficacy is primarily contingent upon the generation of Abs that protect against infection. Protective Abs mediate pathogen control and clearance by neutralization and Fc effector functions. In some cases, however, pathogen-specific Abs can enhance disease, as seen during re-infection with dengue virus (1, 2).

The development of an HIV vaccine capable of eliciting protective Abs remains one of the highest priorities of the HIV/AIDS research effort. While neutralizing Abs can prevent infection and suppress HIV/SIV replication in humans and Indian rhesus macaques (RMs), high levels of neutralizing Ab titers against a neutralization resistant tier 3 virus not yet been induced by vaccination. However, nearly all individuals produce non-neutralizing Abs after infection with HIV, and many vaccination regimens have induced such non-neutralizing, Env-binding Abs. The scientific community is still evaluating the role of non-neutralizing Abs in HIV/SIV infection. The results of adenovirus 26 (Ad26) vaccination and transfer studies in RMs (3–5) suggested the potential benefit that non-neutralizing Env-binding Abs might have against HIV/SIV. Interestingly, protection in Ad26-vaccinated RMs was associated with the induction of polyfunctional Abs with the capacity to mediate Fc effector functions, including Ab-dependent cellular cytotoxicity (ADCC), Ab-dependent cellular phagocytosis (ADCP), and Ab-dependent complement deposition (ADCD) (3, 4).

Control of HIV/SIV replication after infection has been previously described. Some HIV-infected individuals and SIV-infected RMs expressing certain major histocompatibility complex class I (MHC-I) alleles spontaneously control viremia during early chronic infection in the absence of antiretroviral therapy. Recently, we reported an unusual group of immunized Mamu-B^*^17+ RMs that manifested stringent control of the highly pathogenic SIVmac239 infectious molecular clone after being vaccinated with *vif*, *nef* and *env* (6). While around 20% of unvaccinated Mamu-B^*^17+ RMs naturally control SIVmac239 replication, it usually takes 12–20 weeks to reduce viral loads below 1,000 viral RNA (vRNA) copies/ml (7). In this study, five of the eight env-vaccinated Mamu-B^*^17+ RMs suppressed viral replication to undetectable levels (<15 vRNA copies/ml) by 4–8 weeks post-infection. Remarkably, Mamu-B^*^17+ RMs vaccinated with *vif* and *nef* only (without *env*) did not exhibit this stringent level of control of SIVmac239 replication, implicating vaccine-induced Env-specific immune responses in control of replication (6). Indeed, control was associated with high endpoint titers of vaccine-induced gp140-binding Abs on day of challenge. Interestingly, these vaccine-induced Abs did not neutralize SIVmac239, and serum ADCC activity did not correlate with control. This level of control resembled the rapid and profound control of SIVmac239 replication manifested by RMs vaccinated with the 68-1 rhesus cytomegalovirus (rhCMV) expressing SIV proteins (8–10).

To further investigate the mechanism of virologic control in our Mamu-B^*^17+ rapid controllers, we designed a multifaceted study aimed at determining the relative contribution of vaccine-elicited non-neutralizing Abs to the rapid viremic control observed in this unique RM cohort. Using systems serology (11), we first characterized the functional properties of these vaccine-elicited Env-binding Abs. To determine if these Abs contributed to suppression of viral replication, we monitored viral loads of two Mamu-B^*^17+ rapid controllers with viral loads below limits of detection after isolating and depleting circulating Abs by immunoadsorption and administration of a neonatal Fc receptor (FcRn)-blocking Ab. Finally, we evaluated the efficacy of these Abs *in vivo* by administering the purified immunoglobulin (Ig) from the two rapid controllers to six vaccinated RMs 1 day prior to intrarectal SIVmac239 challenge. Collectively, our data suggest that non-neutralizing Abs may contribute to control of SIVmac239 replication *in vivo*.

## Results

### Characterization of Circulating SIVmac239-Specific Abs in Immunized Mamu-B^*^17+ Indian Rhesus Macaques

**Figure 1:**
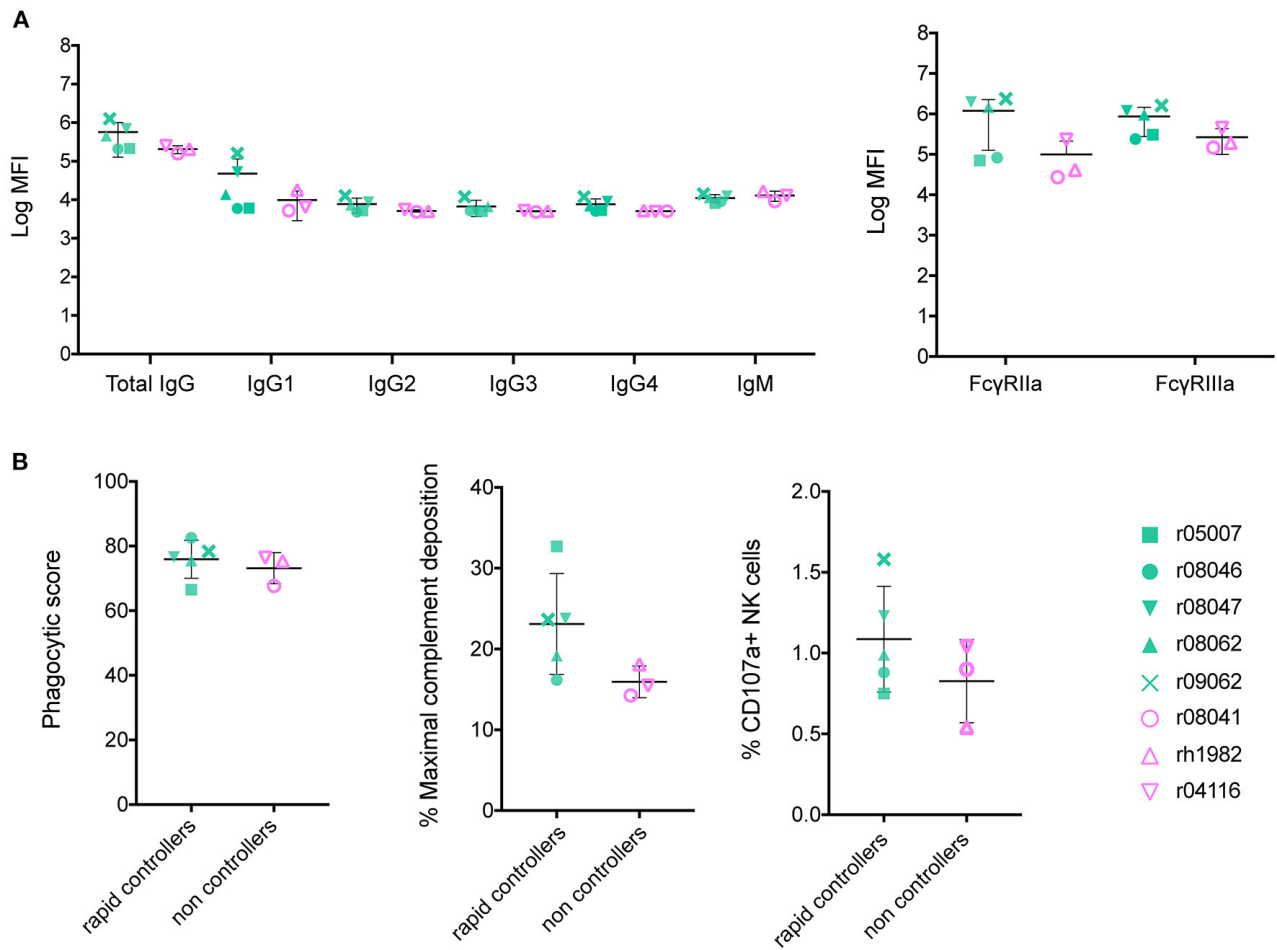
Characterization of Env-binding Abs from Mamu-B*17+ RMs at the time of first SIV challenge. Sera of immunized Mamu-B*17+ rapid controllers (in green) were compared to sera of non-controllers (in pink) prior to challenge at 1:500 dilution. **(A)** gp140-specific Ab subclass and isotype titers, and rhesus FcγRIIa/FcγRIIIa affinity were measured by Luminex. Mean log MFI for negative controls was 3.7. **(B)** gp140-specific responses for each of the three Ab effector functions measured: ADCP, ADCD, and NK degranulation. None of the differences between rapid controllers and non-controllers at the time of SIV challenge from both panels were statistically significant (*p* > 0.05 by Mann-Whitney *U* test).

To identify a shared Ab signature among the five vaccinated rapid controllers from our previous vaccine trial, we assessed the vaccine-induced Ab responses developed in each animal at the time of the first challenge by systems serology (12). We had previously determined SIVmac239 neutralization titers and ADCC activity in these animals (6). Here, we analyzed Ab titers, FcγR affinity, and assessed three Fc-mediated effector functions: ADCP, NK cell degranulation, and ADCD (Figure 1). Because all Fc effector function assays were performed using human cells, rather than rhesus, we first confirmed that these circulating Abs bound human FcγRIIa, FcγRIIb, and FcγRIIIa (Supplementary Figure 1). In the group of eight Env-vaccinated Mamu-B^*^17+ animals, we compared RMs that showed stringent control (*n* = 5) to those that did not (*n* = 3). Rapid controllers had slightly higher total IgG and IgG1 serum concentrations of Abs targeting SIV gp140, although this difference was not statistically significant. These Env-specific Abs also exhibited a higher affinity for rhesus FcγRIIa and FcγRIIIa relative to non-controllers (Figure 1A), which was also not statistically significant. Since Barouch et al. reported that protected Ad26-vaccinated RMs had developed highly polyfunctional Abs (3), we evaluated the capacity of our vaccine-induced Abs to mediate ADCP, ADCD and NK cell activation (Figure 1B). While we detected enhanced ADCD and NK cell degranulation activity (using staining for CD107a) mediated by the gp140-binding Abs present in the serum of the rapid controllers at day of challenge, these differences were not statistically significant.

### Isolation and Depletion of Circulating IgG by Immunoadsorption

**Figure 2:**
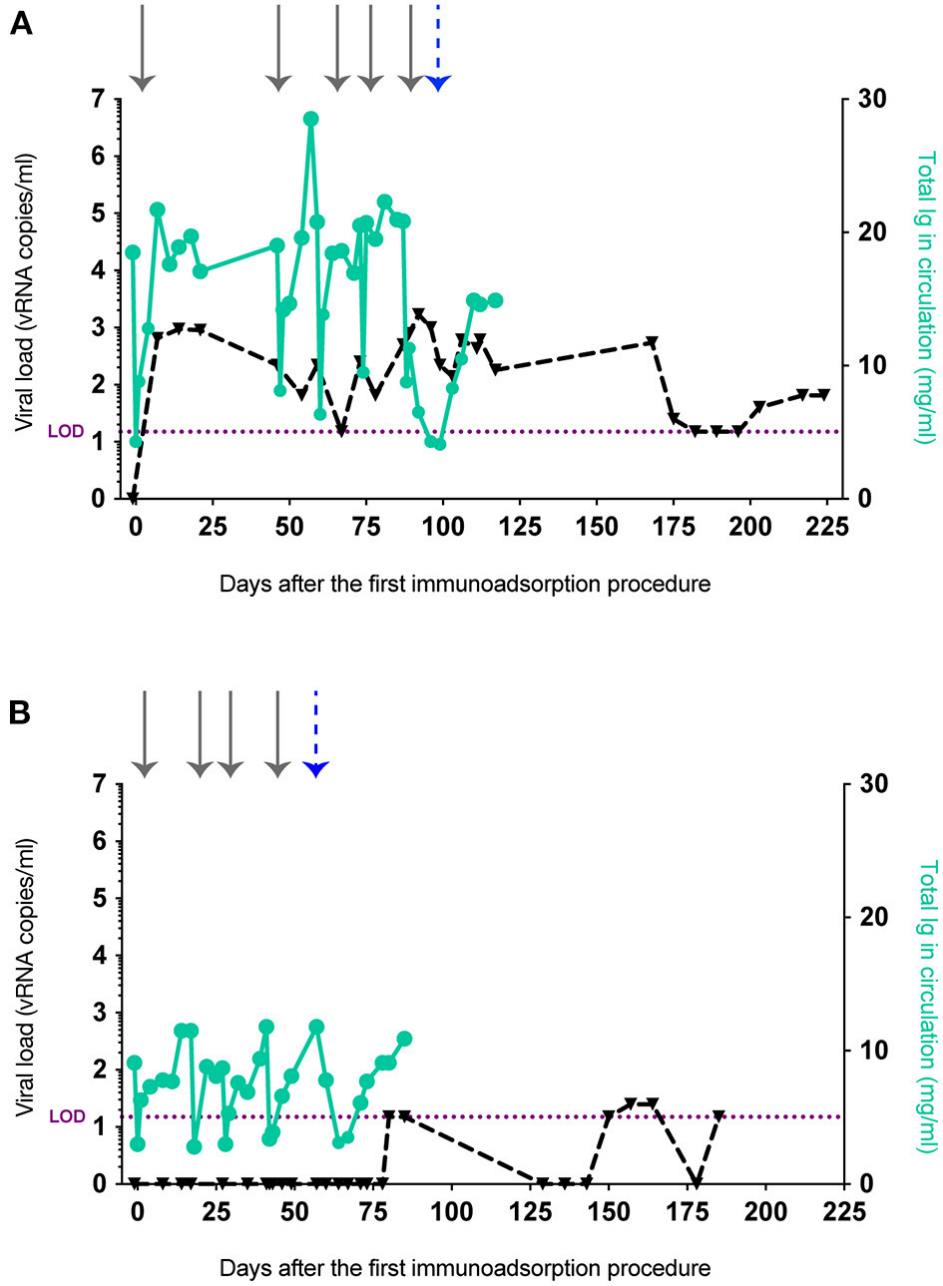
IgG levels and viral loads after the removal of circulating Abs from r05007 and r09062. Ig depletion by immunoadsorption and anti-FcRn Ab infusion of **(A)** r05007 and **(B)** r09062. Gray arrows indicate each immunoadsorption procedure. The blue dashed arrow indicates the first anti-FcRn Ab infusion. Viral load limit of detection (LOD) is shown as purple dotted line.

Next, we explored the hypothesis that the stringent viremic control observed in our previous vaccine trial was mediated by vaccine-induced Abs. We evaluated the ability of these Abs to facilitate control of viral replication in vaccinated animals and whether depletion of these Abs in the stringent controller Mamu-B^*^17+ RMs would result in viral rebound. While it is possible to isolate Ig from serum or plasma by collecting blood draws, the monthly blood available is limited by the weight of each animal. Thus, to overcome this restriction and isolate sufficient Ig to eventually transfer it to another set of vaccinated animals, we subjected r05007 and r09062, the two rapid controllers with the highest Env-specific Ab titers, to at least four rounds of therapeutic plasma exchange with a secondary plasma device using the Spectra Optia system (Terumo BCT) (13). This method has been previously used to remove circulating immunocomplexes and pathological auto-Abs in circulation (14–18). In contrast to plasmapheresis or standard therapeutic plasma exchange, where plasma is removed and replaced with fluids containing albumin and coagulation factors, we employed a continuous-flow centrifugation procedure, also known as immunoadsorption, to selectively remove Ig from plasma before reinfusing the Ig-depleted plasma back into the same animal. Immunoadsorption minimizes the side effects usually associated with plasmapheresis and allowed us to run one liter of plasma, corresponding to 2.5 times the animal's total blood volume, through four protein A or protein G columns. Using this technique, we isolated 15 g of total Ig from r05007 and r09062 over 3 months. After each round of immunoadsorption, IgG levels in both RMs were reduced by 53–76% compared to baseline (defined as the serum IgG concentration before each procedure) (Figures 2A,B). Additionally, we monitored the viral loads of these animals. At the time of the first procedure, both RMs were suppressing viral replication to <15 vRNA copies/ml (Figures 2A,B). Seven days after the first immunoadsorption, r05007 had a viral load of 650 vRNA copies/ml, and on day 14, viremia peaked at 940 vRNA copies/ml (Figure 2A). Viral loads in r05007 fluctuated between 15 and 940 vRNA copies/ml during all five rounds. r09062 maintained undetectable viral loads, <15 vRNA copies/ml, throughout all four procedures (Figure 2B). Thus, the temporary reduction of circulating Abs caused by immunoadsorption led to a transient rebound in SIV replication.

### Anti-neonatal FcR Ab Treatment Depletes Plasma IgG in RMs

FcRn has been described as the main factor responsible for prolonging IgG half-life by protecting it from the lysosomal degradative pathway (19–22). IgG recycling is possible because the FcRn binds to the Fc portion of IgG with high affinity at low pH, but not at neutral pH (23, 24). The pH dependence of the FcRn-IgG interaction facilitates the translocation of IgG from acidic endosomes to the cell surface, then its subsequent release into the near-neutral pH of circulation. Hence, it is not surprising that *in vivo* FcRn dysfunction has been linked to low plasma IgG concentrations (25). Recently, Smith et al. used Rozanolixizumab, an anti-FcRn monoclonal Ab, to safely and efficiently block FcRn and deplete circulating IgG in cynomolgus macaques (26). With this in mind, we reasoned that a prolonged reduction in IgG levels would result in loss of viremic control if the non-neutralizing Abs circulating in the Mamu-B^*^17+ rapid controllers had a role in suppressing SIVmac239 replication. We administered a simianized Rozanolixizumab chimera (anti-FcRn rhIgG1 [RozR1LALA]) to the two rapid controllers. Monkey r05007 received an intravenous dose of 30 mg/kg followed by a subcutaneous daily dose of 5 mg/kg for 1 week, while r09062 received a single intravenous dose of 300 mg/kg. We then quantified circulating IgG and SIVmac239 viral loads. r05007 received the anti-FcRn Ab infusion two days after the last round of immunoadsorption. At the time of the first anti-FcRn Ab administration, the IgG concentration in this animal was already reduced by 45.8% relative to baseline, and viremia had increased from 500 to 790 vRNA copies/ml. Three days after commencing Ab infusion, viral loads peaked at 1,700 vRNA copies/ml. Maximal IgG depletion, an 80.3% decrease relative to baseline, was observed 10 days following the first infusion (or three days after the final subcutaneous dose) (Figure 2A). Unlike r05007, r09062 controlled viral replication throughout all four rounds of immunoadsorption. Although these two Mamu-B^*^17+ animals had been infected for over five years (Supplementary Figure 2), r09062 had maintained viral loads below detection limits for over 4.5 years (Supplementary Figure 2B). Two weeks after the last immunoadsorption, r09062 received a single infusion of anti-FcRn Ab and its IgG levels started to decrease. One week later, IgG levels were reduced 73.9% relative to baseline (Figure 2B). Viral loads remained <15 vRNA copies/ml for 27 days after the anti-FcRn Ab administration. We then started to observe plasma viral “blips” ranging from 15 to 25 vRNA copies/ml over the following 100 days. It should be noted that both animals developed moderate titers of anti-drug Abs after treatment with anti-FcRn Ab, independent of the infusion route and dose (Supplementary Figure 3).

### Ig Characterization and Passive Transfer Study

**Figure 3:**
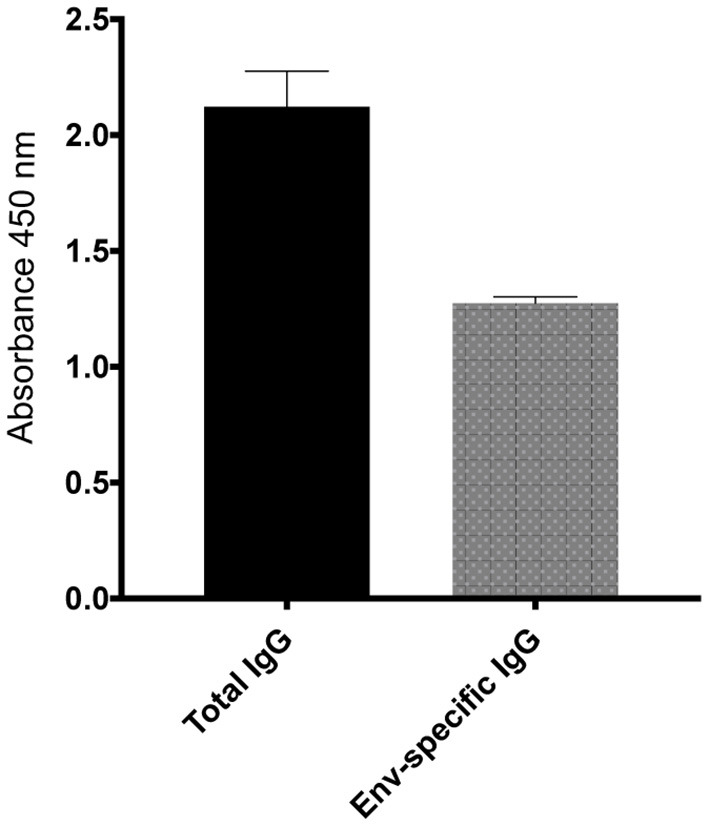
Total and Env-specific binding Abs present in the purified total Ig isolated from the Mamu-B*17+ RMs at 1 μg/ml. At a normalized concentration of 1 μg/ml, a large proportion of Abs present in the purified total IgG bound to SIVmac239 gp140 protein. The purified total Ig had no neutralization activity against SIVmac239 (data not shown).

**Figure 4:**
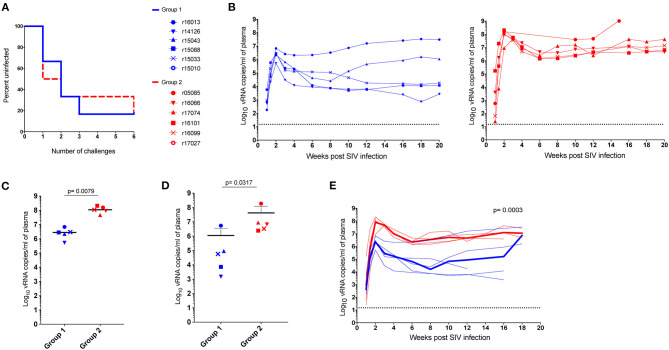
Outcome of SIV challenge. Two years after vaccination, vaccinees and controls were intrarectally challenged with a low dose of SIVmac239. **(A)** Kaplan-Meier analysis of SIV acquisition, **(B)** individual viral loads of Group 1 vaccinees (blue) and the Group 2 (red) RMs. While the difference in acquisition rate was not statistically significant (*p* > 0.05 by log-rank test), **(C)** peak, and **(D)** setpoint viremia were significantly lower for vaccinees than controls (*P* = 0.0079 and *P* = 0.0317, respectively). Peak viral loads were defined as the highest viral load measurement within the first 4 weeks post-infection. Chronic phase setpoint was calculated as the geometric mean of viral load values between 8 and 12 weeks post-infection. **(E)** Geometric mean viral loads for Group 1 and Group 2 animals (*P* = 0.0003). Of note, we did not identify any correlation between the level of Env-binding Abs on the challenge day and take of the infection. *P*-values were determined using the Mann-Whitney *U* test.

To further investigate the virologic control observed in the Mamu-B^*^17+ RMs of our previous vaccine trial, six RMs expressing Mamu-A^*^01+ and/or Mamu-A^*^02+ MHC-I alleles (Table 1) were vaccinated with *gag, nef*, *tat, rev, vif*, *vpr*, and *vpx*. SIV genes were delivered using a recombinant vesicular stomatitis virus (rVSV) prime followed by a recombinant adenovirus type 5 (rAd5) boost. Both vaccine vectors utilized in this study included *gag, tat* and *nef*, or *tat* fused with other genes (i.e., *vif*, *vpr, vpx*, and *rev*) but did not include *env*. As a reference, our Mamu-B^*^17+ cohort was vaccinated with *env, vif*, *nef*, *tat*, and *rev*, which were delivered using a recombinant yellow fever virus 17D prime followed by three boosts with recombinant DNA plasmids delivered by electroporation (6). These animals were then administered rAd5, rVSV and rhesus monkey rhadinovirus (rRRV) constructs, and were challenged almost 4 months after the last rRRV boost. At the time of the first SIV challenge, we detected SIV-specific T cell responses by intracellular cytokine staining (ICS) in the peripheral blood mononuclear cells (PBMCs). These animals had developed vaccine-induced CD8^+^ T cells mainly directed against *vif* and *nef*, and CD4^+^ T cells against *env*. Conversely, we were unable to detect SIV-specific T cell responses, by ICS, in the PBMCs of our Group 1 vaccinees at the time of the first intrarectal SIV challenge (data not shown), which was 2 years after the rAd5 boost. One day before the first SIV challenge, all vaccinated RMs (Group 1) received 200 mg/kg of the purified Ig derived from the two Mamu-B^*^17+ vaccinated animals. A large proportion of the infused Ig targeted SIV Env (Figure 3). At the time of SIV challenge, detectable levels of Env-binding Abs were present in all recipient RMs. Ab levels were comparable among all Group 1 animals and ranged from 221 to 321 μg/ml of gp140-specific IgG in circulation (Table 2). No neutralization activity against SIVmac239 was detected in the purified total Ig (data not shown). We evaluated the contribution of these Abs to protection against acquisition and suppression of viremia post-infection by subjecting all animals to six repeated intrarectal challenges with a marginal infectious dose of SIVmac239, as previously described (27), starting one day following the Ig passive transfer. Six additional unvaccinated, non-Ig-infused RMs expressing Mamu-A^*^01+ and/or Mamu-A^*^02+ MHC-I alleles (Group 2) served as controls for this challenge experiment (Table 1). In Group 1, Env-specific Ab titers decreased by 35% during the first week after infusion and continued to decline until the sixth challenge. Eleven weeks later, we detected 2.25 μg/ml of gp140-specific Abs in r15010, the only RM from Group 1 that remained uninfected after six SIVmac239 challenges (Table 2). Of note, r15010 was the only RM in Group 1 or 2 that also expressed the elite control-associated Mamu-B^*^17+ allele (Table 1). We did not observe any correlation between the level of Env-binding Abs in the sera of the Group 1 vaccinees and the rate of SIVmac239 acquisition. Although vaccinees and control animals acquired SIV at similar rates (Figure 4A), Group 1 exhibited significantly lower peak (*P* = 0.0079) and chronic phase viral loads (*P* = 0.0317) (Figures 4B–D). The difference in geometric mean viremia between both groups was also statistically significant (*P* = 0.0003) (Figure 4E). Collectively, these data suggest that non-neutralizing Abs may contribute to suppression of SIVmac239 replication *in vivo*.

**Table 1:** Animal details.

**Experimental group**	**Animal identifier**	**Relevant MHC-I allele(s)**	Age (years)^*^	**Sex**
Rapid controllers	r05007	Mamu-B^*^17	15	Female
	r09062	Mamu-B^*^17	10.5	Female
Group 1 (Vaccinees)	r15088	Mamu-A^*^01, -A^*^02	4.7	Male
	r16013	Mamu-A^*^02	4.4	Male
	r15033	Mamu-A^*^02	5.0	Male
	r15043	Mamu-A^*^01	4.9	Female
	r15010	Mamu-A^*^02, -B^*^17	5.5	Male
	r14126	Mamu-A^*^01, -A^*^02	5.7	Male
Group 2 (Controls)	r16101	Mamu-A^*^01	3.8	Male
	r17027	Mamu-A^*^01	3.2	Male
	r17074	Mamu-A^*^01	2.9	Male
	r16066	Mamu-A^*^02	4.1	Male
	r16099	Mamu-A^*^02	3.8	Female
	r05085	Mamu-A^*^02	14.8	Female

* *Animal age at the beginning of the study*.

**Table 2:** Env-specific serum IgG (μg/ml) in Group 1 prior to infection.

**Weeks after Ig infusion**	**r15043**	**r16013**	**r14126**	**r15088**	**r15033**	**r15010**	**Average**
0	258.65	263.26	318.87	221.24	321.13	302.54	280.95
1			145.27	107.95	272.92	198.31	181.11
2			54.04	64.09	83.00	80.06	70.30
3					53.50	66.59	60.05
4					43.18	61.56	52.37
5						59.28	59.28
6						29.14	29.14
7						17.40	17.40
8						5.27	5.27
9						2.98	2.98
10						2.52	2.52
11						2.25	2.25

## Discussion

In this study, we assessed the potential role of vaccine-elicited non-neutralizing Abs in our Mamu-B^*^17+ rapid controllers in the prevention and control of SIV infection. First, we characterized the functional profile of the vaccine-induced Abs from our vaccinated Mamu-B^*^17+ cohort, which demonstrated stringent control of SIVmac239 replication (6). Next, we isolated and depleted circulating Ig from two Mamu-B^*^17+ rapid controllers that suppressed viral replication to below detection limits for an extended period of time. The removal of Ig from serum of these two elite controllers resulted in transient low levels of measurable viremia in both RMs. To determine whether the hyperimmune anti-SIV Ig fraction isolated from the Mamu-B^*^17+ RMs could contribute to suppression of viral replication in animals lacking elite control-associated MHC-I alleles, we infused this pooled total Ig into six vaccinated RMs one day before intrarectal SIVmac239 challenge. While passive transfer of total Ig did not protect against infection, the vaccinees exhibited significant reductions in peak and chronic phase viremia relative to the control group. Overall, these data suggest that non-neutralizing Abs may play a role in SIVmac239 suppression *in vivo*.

Our previous analysis suggested that Env-specific non-neutralizing Abs could contribute to the stringent control of SIVmac239 observed in the Mamu-B^*^17+ rapid controllers (6). However, the mechanistic basis for rapid control remains unknown. In the present study, we evaluated whether Fc-mediated effector functions were involved in such stringent suppression. Although none of these functions correlated with rapid control, the Fc domain is known to engage a wide spectrum of receptors on different cell types of the immune system. Bournazos et al. recently evaluated the effect of FcγR binding of influenza-specific Abs in protecting humanized mice against lethal influenza challenge (28–30). Mice treated with these Abs showed enhanced protection when the Fc region was modified to engage FcγRIIa, alone or in combination with FcγRIIIa (31, 32). Further, the authors did not observe antiviral protection in FcγR-deficient mice treated with these influenza-specific Abs (33). It remains possible that the mechanistic basis for rapid control in Mamu-B^*^17+ RMs involves binding of these non-neutralizing Abs to FcγRs. Antigen-Ab immune complexes, for example, could have elicited SIV-specific CD8^+^ T cell responses in those animals via engagement of antigen-presenting cells, perhaps driving dendritic cell maturation and antigen cross-presentation.

Mamu-B^*^17+ RMs are naturally predisposed to spontaneously control SIV infection without antiretroviral therapy. Previous studies have implicated Mamu-B^*^17-restricted CTLs as the main contributors to elite control in these RMs (6). However, the contribution of SIV-specific Abs to this elite control is unclear. Recently, we vaccinated two groups of Mamu-B^*^17+ RMs with *vif* and *nef*, with and without the addition of *env*. Interestingly, the group that received *env, vif*, and *nef* showed unprecedented rapid control of SIVmac239 after infection, highlighting the potential role of vaccine-induced, non-neutralizing Abs in suppressing viremia. One of the many advantages of the SIV-infected RM model is the ability to determine the contribution of a particular cell type by depleting it and measuring viral rebound (34–39). Currently, it is possible to ablate cells *in vivo* by using depleting monoclonal Abs that target a particular cell type. We and other groups have previously depleted CD8β^+^ T cells and CD20^+^ cells with the anti-CD8β Ab CD8β255R1 (38–40) and anti-CD20 Ab Rituximab (41–43), respectively. Although Rituximab can efficiently deplete CD20^+^ cells from circulation and lymphoid tissue (42), Ab-secreting cells in RMs have surface marker phenotypes that differ from those of humans, making them challenging to identify and adequately deplete. Since there are no existing regimens for depletion of Ab-secreting cells in RMs, we aimed to reduce levels of circulating Abs in our SIV-infected Mamu-B^*^17+ RMs through two novel approaches: immunoadsorption and the infusion of an anti-FcRn Ab. These strategies depleted up to 80% of serum Abs in r05007 and r09062. Previous studies have demonstrated that near-complete depletion of circulating CD8β^+^ T cells can permit transient viral rebound in elite controllers (38, 39). Because we were unable to completely deplete circulating Abs in our animals, it is possible that the remaining Abs in circulation mitigated viral rebound, thereby “masking” their potential involvement in viremic suppression. These “blips” of transient viremia in both Mamu-B^*^17+ RMs suggest that the removal of Env-binding Abs partially contributed to such control. However, since the total amount of Abs in circulation regulates frequencies of activated B cells and the total quantity of Ig produced through a quorum sensing-like mechanism (44), our experimental interventions may have resulted in inadvertent B cell activation and stimulation of latently-infected CD4^+^ T cells in lymphoid follicles, thereby resulting in the transient viremia observed in these animals. Thus, we cannot definitively conclude that the depletion of non-neutralizing Env-binding Abs from circulation directly permitted viral rebound, and additional studies will be required to determine the true efficacy of these Abs in suppressing viral replication *in vivo*.

In this study, we attempted to determine whether vaccine-induced Env-specific Abs could mediate stringent viremic control in Mamu-B^*^17-negative RMs. We passively transferred purified Ig from two rapid controllers to six vaccinated RMs that expressed the Mamu-A^*^01+ and/or Mamu–A^*^02+ MHC-I alleles. We demonstrated that this vaccine regimen, along with Ab infusion, resulted in statistically significant reductions in both peak and chronic phase viral loads in the vaccinees. Previously, we have shown that Mamu-A^*^01+ and/or Mamu-A^*^02+ RMs immunized with *nef, tat, vif*, *rev*, and either *env, gag*, or both, have significantly lower peak viral loads than unvaccinated control animals (45). However, control of chronic phase viremia was only observed in the group of animals that were vaccinated with six of the viral genes (*env, gag, vif, rev, tat*, and *nef*). Notably, the vaccine-induced Abs detected in these *env*-vaccinated animals did not neutralize SIVmac239. All other groups failed to control chronic phase viral replication. The animals in the present study were only vaccinated with *gag, vif, rev, tat*, and *nef* (and not *env*), and based on these previous results, would not be expected to control chronic phase viremia. It can be presumed, therefore, that Ig infusion of the Group 1 vaccinees resulted in better control than might be expected in animals vaccinated with only *gag, vif, rev, tat*, and *nef*. Interestingly, the RMs described in this paper were challenged two years after vaccination and had no detectable Gag-specific CD8^+^ T cells in their PBMCs. Nevertheless, low levels of vaccine-induced Gag-specific CD8^+^ T cells (undetectable by ICS) could still be present in these animals. These CD8^+^ T cells could have been stimulated by Ab immune complexes and antigen-presenting cells after infection, and then contribute to the suppression of SIVmac239 replication, similarly to what could have occurred in our Mamu-B^*^17+ rapid controllers.

Other groups have protected RMs from SIV and simian-human immunodeficiency virus (SHIV) acquisition by administering neutralizing Abs (46–49). Compared to non-neutralizing Abs, neutralizing Abs have been shown to be effective at low titers. Moldt et al. protected RMs against SHIV after infusing 5 and 1 mg/kg of PGT121 (46), a potent broadly neutralizing monoclonal Ab, one day before mucosal challenge. Not surprisingly, the ability to neutralize the challenge virus appears to be a key factor in preventing SIV/SHIV acquisition. Neutralizing Abs can be present at much lower concentrations in the target tissue and still be able to confer protection. Although the purified Ig was administered intravenously, both Group 1 and 2 RMs of this study were challenged with an intrarectal marginal infectious dose of SIVmac239. It has been previously shown that Ig tissue distribution is slow and determined by the rate and extent of blood perfusion into the target tissue. In fact, Schneider et al. used fluorescent Abs to determine Ig anatomical distribution after infusion in non-human primates (50). In that study, the authors collected plasma, tissue biopsies and mucosal secretions up to eight weeks following the administration of these Abs. Interestingly, Ab levels in plasma peaked after 24 h, but sequential biopsies showed Ab loading in the vaginal and rectal mucosa up to one week after infusion. Accordingly, the Env-specific Abs in our Group 1 vaccinees would likely not have perfused the rectal mucosa at stable levels until 72 h after infusion, two days after the first SIV challenge. Conversely, Alter et al. recently published a study where they protected naïve RMs after an intravenous infusion of vaccine-induced non-neutralizing Abs, at a dose of 100 mg/kg, one day before intrarectal SIVmac251 challenge (5). Thus, it is possible that a particular functional profile and concentration of non-neutralizing Abs in the target tissue is needed to protect against intrarectal SIV challenge.

In summary, we investigated the role of non-neutralizing Abs in the prevention and control of SIVmac239 replication. To do this, we developed two methodologies to isolate and deplete circulating Abs from RM sera, and transferred the isolated total Ig from our rapid controllers to another group of vaccinated RMs. While we detected low levels of transient viremia in the two Mamu-B^*^17+ RMs, additional studies with greater levels of Ig depletion might be necessary to elucidate the true contribution of non-neutralizing Abs to viremic suppression in elite controllers. In our passive transfer study, we did not prevent SIVmac239 acquisition, even after delivering large amounts of purified Ig. Interestingly, while *gag*-vaccinated RMs effectively reduce peak, but not chronic phase viral loads, the administration of Env-binding Abs to these animals significantly reduced chronic phase viremia. Together, our data indicate that Env-binding Abs can potentially contribute to control SIVmac239 replication *in vivo*, independently of MHC-I genotype. However, larger monkey studies will be needed to validate this hypothesis.

## Materials and Methods

### Animal Experiments

The 14 Indian RMs (*Macaca mulatta*) used in this study were housed at the Wisconsin National Primate Research Center (WNPRC). All animals were cared for in accordance with Weatherall report guidelines and the recommendations included in the National Research Council Guide for the Care and Use of Laboratory Animals. All procedures were approved by the University of Wisconsin Graduate School Animal Care and Use Committee (animal welfare assurance number 16-00239 [A3368-01]; protocol number G005248). RMs were separated as follows: Group 1 (Ig-infused vaccinated RMs, *n* = 6), Group 2 (controls, *n* = 6), and Group 3 (Mamu-B^*^17 rapid controllers, *n* = 2). Purified Ig was prepared in saline bags and administered intravenously into each animal. The rhesus IgG1 chimeric version of Rozanolixizumab, Anti-FcRn Ab [RozR1LALA], was engineered and produced by the nonhuman primate reagent resource (NIH Nonhuman Primate Reagent Resource Cat# PR-00-1, RRID: AB_2888630). Anti-FcRn Ab, which was infused in Group 3 animals, was initially administered intravenously, followed by a subcutaneous daily dose for a week in one of the Group 3 RMs. We collected plasma and serum at the indicated time points to measure viremia, total Ig and Env-specific Ab levels.

### Multiplex gp140-Specific Ig Subclass and Fc Binding Affinity Assay

To determine the relative concentrations of total IgG, IgG1, IgG2, IgG3, IgG4, and IgM in the sera of the Mamu-B^*^17+ RMs at the time of the first SIV intrarectal challenge, we first coupled SIVmac239 gp140 to magnetic beads (Luminex Corporation) using NHS-ester linkages with Sulfo-NHS and EDC (Thermo Fisher). Next, we incubated SIVmac239 gp140-conjugated magnetic beads with diluted serum overnight at 4°C on a shaker. The following day, plates were spun down and washed before adding these reagents to the appropriate wells: mouse anti-monkey IgG PE (SouthernBiotech, #4700-09), to determine total IgG, mouse anti-rhesus Ig (rhIg) G1 (NHPRR, clone 7H11), rhIgG2 (NHPRR, clone 3C10), rhIgG3 (NHPRR, clone 2G11), rhIgG4 (NHPRR, clone 7A8), and rhIgM (Life Diagnostics, #2C11-1-5), to define the relative concentration of each isotype, or PE-conjugated FcγRIIa, FcγRIIb, and FcγRIIIa (R&D Systems, #1330-CD-050, #1875-CD-050, #4325-FC-050, respectively). The plates were incubated for 1 h at room temperature on a shaker. Next, we added goat anti-mouse IgG Fc PE (Invitrogen, #31861) to the corresponding “isotype” wells and incubated the plates for 1 h at room temperature on a shaker. After washing all plates, fluorescence was quantified using an IQue 3 flow cytometer (Intellicyt) and analysis was performed using IntelliCyt ForeCyt (v8.1).

### Antibody-Dependent Cellular Phagocytosis

A THP-1 phagocytosis assay was performed using SIVmac239 gp140-coated yellow/green fluorescent-neutravidin beads (Molecular Probes Inc, #F8776), as previously described (51). First, the fluorescent beads were incubated with diluted plasma for 2 h at 37°C. Following this incubation, THP-1 cells were added to the plates and incubated at 37°C overnight to allow phagocytosis. The next day, cells were fixed and acquired using a LSRII flow cytometer (BD Biosciences). Pooled plasma samples were first titrated and then individually tested at a final dilution of 1:500. We calculated the phagocytic scores as the geometric mean fluorescent intensity (MFI) of the FITC-fluorescent beads multiplied by the bead uptake percentage.

### Antibody-Dependent Complement Deposition

ADCD was assessed by monitoring C3b complement deposition using SIVmac239 gp140-coated red fluorescent neutravidin beads (Molecular Probes Inc, #F8775). Beads were first incubated with diluted plasma for 2 h at 37°C. After washing the bead/plasma mixture, we added guinea pig complement (Cedarlane Laboratories, #CL4051), diluted in gelatin veronal buffer with calcium and magnesium (Boston Bioproducts), and incubated the plates for 20 min at 37°C. Following this incubation, all wells were washed with cold 15 mM EDTA, then incubated with FITC-conjugated goat anti-guinea pig C3 Ab (MP Biomedicals, #855385) for 15 min at room temperature. Last, the plates were washed, and beads were resuspended in PBS. Samples were acquired using a LSRII flow cytometer (BD Biosciences). Percentage of complement deposition was determined by the geometric MFI multiplied by the percentage of beads positive for C3b deposition.

### Antibody-Dependent NK Cell Activation

Plates were coated with SIVmac239 gp140 and incubated overnight at 4°C. All coated wells were washed and blocked with 5% BSA in PBS for 1 h at 37°C. Plates were then washed and diluted plasma samples were added into the corresponding wells for 1 h at 37°C. Simultaneously, CD107a PE Cy5 (BD Biosciences, #555802), brefeldin A (Sigma, #B7651), and GolgiStop (BD Biosciences, #554724) were incubated with human NK cell culture for 2 h. After both incubations were completed, we added the stimulated NK cells to the plates (50,000 cells per well), and incubated them for 5 h at 37°C. Next, NK cells were transferred to V-bottom plates containing CD56 PE Cy7 (BD Biosciences, # 557747), CD16 APC Cy7 (BD Biosciences, #55775), and CD3 Alexa Fluor 700 (BD Biosciences, #557943) and incubated for 15 min at room temperature. Plates were then washed and resuspended in PBS. Samples were acquired using a LSRII (BD Biosciences). Human NK cells were isolated using a RossetteSep NK cell enrichment kit (StemCell Technologies, #15065). NK cells were defined as CD3^−^/CD16^+^ and/or CD56^+^. Ab-dependent NK degranulation was calculated as the percentage of NK cells positive for CD107a.

### Anti-Env Antibody Enzyme-Linked Immunosorbent Assay (ELISA)

The characterization and *in vivo* half-life of the purified Ig isolated from the Mamu-B^*^17+ RMs were determined by ELISA. ELISA plates were coated with either purified mouse anti-human Ig (BD Biosciences, #555784) or purified SIVmac239 gp140 (Immune Technologies, #IT-001-140p) at 1 μg/ml and incubated overnight at 4°C. The next day, all plates were washed with 1 × PBS-Tween20 and blocked with 5% powdered milk in PBS at 37°C for 1 h. Following this incubation, the plates were washed and 100 μl of serially diluted serum samples or purified Mamu-B^*^17-derived total Ig was added to the corresponding wells for 1 h at 37°C. After washing the plates, 100 μl of diluted goat anti-human IgG-HRP Ab (SouthernBiotech, #2045-05) was added to all wells and incubated for 1 h at 37°C. The plates were washed before being developed with 3,3′,5,5′-Tetramethylbenzidine (EMD Millipore, #613544-100ML). Shortly after, the reaction was stopped with TMB stop solution (SouthernBiotech, #0412-01) and all plates were read at 450 nm on an Epoch microplate reader (Biotek).

### SIVmac239 Neutralization Assays

We assessed the SIVmac239 neutralization activity of the purified Ig and sera of the Ig-infused RMs using a previously described assay (52). Replication-competent SIVmac239 was produced by transfecting HEK293T cells with the plasmid using JetPrime (Polyplus transfection, #114-01). Seventy-two hours later, supernatant was harvested and stored at −80°C. Purified Ig and RM sera were incubated with SIVmac239 for 1 h at 37°C before being transferred onto TZM-bl cells (NIH AIDS Reagent program, #8129). After 48 h, neutralization activity was calculated based on the luminescence measurements of each duplicate well using the Bright-Glo luciferase assay (Promega, #E2620).

### Vaccination

The Group 1 RMs were vaccinated with genes encoding SIVmac239 *gag, nef*, *tat, rev, vif*, *vpr*, and *vpx* using a rVSV prime followed by a rAd5 boost. The rVSV prime consisted of three plasmids containing the full-length Gag polyprotein (VSV-Gag_1_-G_nj6_), Nef protein (VSV-Nef_1_-G_nj6_), or Tat-Rev fusion protein (VSV-Tat-Rev_1_-G_nj6_). Profectus Bioscience manufactured these vectors. Each Group 1 animal received an intramuscular injection containing 1 × 10^8^ PFU of each VSV vector. Six weeks later, the Group 1 RMs were infused with three rAd5 vectors produced by ViraQuest. These vectors encoded *gag* (rAd5-Gag), *nef* (rAd5-Nef), or a fusion of *vif-vpx-vpr-tat-rev* genes. Each animal received an intramuscular injection containing 1x10^11^ PFU of each Ad5 vector.

### Intracellular Cytokine Staining

PBMCs from Group 1 RMs at the time of the first SIV challenge were stimulated with Gag, Nef and Tat peptides in R10 (RPMI, 10% FBS, 1 × Pen/Strep/Amphotericin B) media containing anti-CD28 and CD49d costimulatory Abs (BD Biosciences), as well as a PE-conjugated anti-CD107a Ab (BioLegend), for 1 h at 37°C in a 5% CO_2_ incubator. Following this incubation, brefeldin A (Sigma, #B7651), and GolgiStop (BD Biosciences, #554724) were added to inhibit protein transport. All tubes were incubated for eight additional hours at 37°C. The antigenic stimuli used for ICS consisted of pools of overlapping 15-mer peptides covering the entire open reading frames (ORFs) of SIVmac239 Gag, Tat and Nef at a final concentration of 1 μM. The following day, PBMCs were washed prior to adding the surface staining Ab cocktail. This cocktail included ARD Aqua (live/dead viability dye) and Abs against: CD4, CD8, CD14, CD16, CD20. Next, the cells were fixed and permeabilized using the Cytofix/cytoperm fixation/permeabilization solution kit (BD Biosciences, #554714). After permeabilization, PBMCs were incubated with Abs against CD3, IFN-γ, TNF-α, and CD69 for 1 h at room temperature. Last, the cells were washed and acquired using a FACSAria II (BD Biosciences). We analyzed the data using FlowJo 10.6 by gating on live CD14^−^/CD16^−^/CD20^−^/CD3^+^ lymphocytes and on cells expressing either CD4 or CD8, but not both markers. Next, we gated cells positive for IFN-γ, TNF-α, or CD107a to determine if they were co-expressed with CD69, a marker of recent lymphocyte activation. We utilized the Boolean gate to analyze all possible combinations. Leukocyte activation cocktail-stimulated PBMCs were stained and used as a positive control.

### SIV Challenge

The challenge stock used in this study was produced by the Virology Services Unit of the WNPRC using SIVmac239 hemi-genome plasmids obtained from the NIH AIDS Research and Reference Reagents Program. Plasmids were transfected into 293T cells. Supernatant containing SIVmac239 virions was harvested and propagated on mitogen-activated rhesus PBMCs. Several healthy SIV negative rhesus macaques were used as donors. The titer of the stock utilized was 90,000 50% tissue culture infective doses (TCID_50_) per ml. All Mamu-A^*^01+ and Mamu-A^*^02+ animals were intrarectally challenged with 200 TCID_50_ (4.8 × 10^5^ vRNA copies) of SIVmac239, as previously described (27). Each challenge occurred every 2 weeks. Plasma samples were taken at 7 and 10 days after exposure to determine if an animal had become infected. Once an animal was SIV positive, it was no longer challenged.

### SIV Viral Load Measurements

SIV RNA viral load measurements were based on a previously published protocol (53). Total RNA was extracted from 0.5 ml of EDTA-anticoagulated Indian RM plasma using QIAgen DSP virus/pathogen Midi kits on a QIASymphony SP instrument. A two-step RT-PCR reaction using random hexamers was completed for each of the six replicates per sample, followed by 45 PCR cycles. The limit of detection in 0.5 ml of plasma was 15 vRNA copies/ml. The primers and probe used for this procedure were: forward primer, SGAG21, 5′ GTCTGCGTCAT(dP)TGGTGCATTC 3′; reverse primer SGAG22, 5′ CACTAG(dK)TGTCTCTGCACTAT(dP)TGTTTTG 3′; probe, PSGAG23, 5′ FAM-CTTC(dP)TCAGT(dK)TGTTTCACTTTCTCTTCTGCG-BHQ1 3′.

### Statistics

Differences between the Mamu-B^*^17+ rapid controllers and non-controllers at day of challenge, as well as viral loads of the Group 1 RMs, were determined using Mann-Whitney *U* test. Kaplan-Meier survival analysis was used to determine if the SIVmac239 acquisition rate differed between both groups. A 0.05 significance threshold was utilized for each test.

## Data Availability Statement

The raw data supporting the conclusions of this article will be made available by the authors, without undue reservation.

## Ethics Statement

The animal study was reviewed and approved by Wisconsin Graduate School Animal Care and Use Committee.

## Author Contributions

DW, NP-L, and MM: conceptualization. DW, NP-L, BR, TV, WF, MG, KC, MR, JL, GA, ER, DM, and MM: writing—review and editing. DW, NP-L, BR, TV, WF, MG, KC, MR, CP, JL, GA, ER, DM, and MM: investigation. DW: resources and supervision. NP-L and MM: methodology. All authors contributed to the article and approved the submitted version.

## Conflict of Interest

JL was employed by Leidos Biomedical Research Inc. MM has a consulting financial interest in Emmune, Inc., a company that is developing HIV immunotherapies based on the immunoadhesin eCD4-Ig. The remaining authors declare that the research was conducted in the absence of any commercial or financial relationships that could be construed as a potential conflict of interest.

## References

[1] Halstead SB. *In vivo* enhancement of dengue virus infection in rhesus monkeys by passively transferred antibody. J Infect Dis. (1979) 140:527–33. 10.1093/infdis/140.4.527 117061

[2] Katzelnick LC Gresh L Halloran ME Mercado JC Kuan G Gordon A. Antibody-dependent enhancement of severe dengue disease in humans. Science. (2017) 358:929–32. 10.1126/science.aan6836 29097492 PMC5858873

[3] Barouch DH Alter G Broge T Linde C Ackerman ME Brown EP. HIV-1 vaccines. Protective efficacy of adenovirus/protein vaccines against SIV challenges in rhesus monkeys. Science. (2015) 349:320–4. 10.1126/science.aab3886 26138104 PMC4653134

[4] Barouch DH Tomaka FL Wegmann F Stieh DJ Alter G Robb ML. Evaluation of a mosaic HIV-1 vaccine in a multicentre, randomised, double-blind, placebo-controlled, phase 1/2a clinical trial (APPROACH) and in rhesus monkeys. Lancet. (2018) 392:232–43. 10.1016/S0140-6736(18)31364-3 30047376 PMC6192527

[5] Alter G Yu WH Chandrashekar A Borducchi EN Ghneim K Sharma A. Passive transfer of vaccine-elicited antibodies protects against SIV in rhesus macaques. Cell. (2020) 183:185–96 e14. 10.1016/j.cell.2020.08.033 33007262 PMC7534693

[6] Martins MA Tully DC Pedreno-Lopez N von Bredow B Pauthner MG Shin YC. Mamu-B^*^17(+) rhesus macaques vaccinated with env, vif, and nef manifest early control of SIVmac239 replication. J Virol. (2018) 92:e00690-18. 10.1128/JVI.00690-18 29875239 PMC6069176

[7] Yant LJ Friedrich TC Johnson RC May GE Maness NJ Enz AM. The high-frequency major histocompatibility complex class I allele Mamu-B^*^17 is associated with control of simian immunodeficiency virus SIVmac239 replication. J Virol. (2006) 80:5074–7. 10.1128/JVI.80.10.5074-5077.2006 16641299 PMC1472056

[8] Hansen SG Ford JC Lewis MS Ventura AB Hughes CM Coyne-Johnson L. Profound early control of highly pathogenic SIV by an effector memory T-cell vaccine. Nature. (2011) 473:523–7. 10.1038/nature10003 21562493 PMC3102768

[9] Hansen SG Piatak M Jr Ventura AB Hughes CM Gilbride RM Ford JC. Immune clearance of highly pathogenic SIV infection. Nature. (2013) 502:100–4. 10.1038/nature12519 24025770 PMC3849456

[10] Hansen SG Marshall EE Malouli D Ventura AB Hughes CM Ainslie E. A live-attenuated RhCMV/SIV vaccine shows long-term efficacy against heterologous SIV challenge. Sci Transl Med. (2019) 11:eaaw2607. 10.1126/scitranslmed.aaw2607 31316007 PMC6788755

[11] Chung AW Alter G. Systems serology: profiling vaccine induced humoral immunity against HIV. Retrovirology. (2017) 14:57. 10.1186/s12977-017-0380-3 29268769 PMC5740944

[12] Chung AW Kumar MP Arnold KB Yu WH Schoen MK Dunphy LJ. Dissecting polyclonal vaccine-induced humoral immunity against HIV using systems serology. Cell. (2015) 163:988–98. 10.1016/j.cell.2015.10.027 26544943 PMC5490491

[13] Kim DK Kim S Jeong SH Kim HO Kim HJ. Therapeutic plasma exchange using the spectra optia cell separator compared with the COBE Spectra. Ann Lab Med. (2015) 35:506–9. 10.3343/alm.2015.35.5.506 26206687 PMC4510503

[14] Sato T Ishigaki Y Komiya T Tsuda H. Therapeutic immunoadsorption of acetylcholine receptor antibodies in myasthenia gravis. Ann N Y Acad Sci. (1988) 540:554–6. 10.1111/j.1749-6632.1988.tb27170.x 3061349

[15] Sato H Watanabe K Azuma J Kidaka T Hori M. Specific removal of IgE by therapeutic immunoadsorption system. J Immunol Methods. (1989) 118:161–8. 10.1016/0022-1759(89)90002-1 2647856

[16] Kiprov DD Simpson DM Pfaeffl W Romanick-Schmiedl S Abrams D Miller RG. AIDS and apheresis procedures–therapeutic and safety considerations. Blood Purif. (1987) 5:51–6. 10.1159/000169456 3790272

[17] Braun N Kadar JG Risler T. Therapeutic immunoadsorption–its role in clinical practice. Transfus Sci. (1998) 19:65–9. 10178698

[18] Schneidewind JM Winkler R Ramlow W Tiess M Hertel U Sehland D. Immunoadsorption–a new therapeutic possibility for multiple sclerosis? Transfus Sci. (1998) 19:59–63. 10.1016/s0955-3886(97)00105-7 10178697

[19] Kim J Hayton WL Robinson JM Anderson CL. Kinetics of FcRn-mediated recycling of IgG and albumin in human: pathophysiology and therapeutic implications using a simplified mechanism-based model. Clin Immunol. (2007) 122:146–55. 10.1016/j.clim.2006.09.001 17046328 PMC2791364

[20] Roopenian DC Akilesh S. FcRn: the neonatal Fc receptor comes of age. Nat Rev Immunol. (2007) 7:715–25. 10.1038/nri2155 17703228

[21] Chaudhury C Mehnaz S Robinson JM Hayton WL Pearl DK Roopenian DC. The major histocompatibility complex-related Fc receptor for IgG (FcRn) binds albumin and prolongs its lifespan. J Exp Med. (2003) 197:315–22. 10.1084/jem.20021829 12566415 PMC2193842

[22] D'Hooghe L Chalmers AD Heywood S Whitley P. Cell surface dynamics and cellular distribution of endogenous FcRn. PLoS ONE. (2017) 12:e0182695. 10.1371/journal.pone.0182695 28817705 PMC5560688

[23] Raghavan M Bonagura VR Morrison SL Bjorkman PJ. Analysis of the pH dependence of the neonatal Fc receptor/immunoglobulin G interaction using antibody and receptor variants. Biochemistry. (1995) 34:14649–57. 10.1021/bi00045a005 7578107

[24] Ward ES Zhou J Ghetie V Ober RJ. Evidence to support the cellular mechanism involved in serum IgG homeostasis in humans. Int Immunol. (2003) 15:187–95. 10.1093/intimm/dxg018 12578848

[25] Roopenian DC Christianson GJ Sproule TJ Brown AC Akilesh S Jung N. The MHC class I-like IgG receptor controls perinatal IgG transport, IgG homeostasis, and fate of IgG-Fc-coupled drugs. J Immunol. (2003) 170:3528–33. 10.4049/jimmunol.170.7.3528 12646614

[26] Smith B Kiessling A Lledo-Garcia R Dixon KL Christodoulou L Catley MC. Generation and characterization of a high affinity anti-human FcRn antibody, rozanolixizumab, and the effects of different molecular formats on the reduction of plasma IgG concentration. MAbs. (2018) 10:1111–30. 10.1080/19420862.2018.1505464 30130439 PMC6291300

[27] Martins MA Tully DC Cruz MA Power KA Veloso de Santana MG Bean DJ. Vaccine-induced simian immunodeficiency virus-specific CD8^+^ T-cell responses focused on a single nef epitope select for escape variants shortly after infection. J Virol. (2015) 89:10802–20. 10.1128/JVI.01440-15 26292326 PMC4621113

[28] DiLillo DJ Tan GS Palese P Ravetch JV. Broadly neutralizing hemagglutinin stalk-specific antibodies require FcgammaR interactions for protection against influenza virus *in vivo*. Nat Med. (2014) 20:143–51. 10.1038/nm.3443 24412922 PMC3966466

[29] Kallewaard NL Corti D Collins PJ Neu U McAuliffe JM Benjamin E. Structure and function analysis of an antibody recognizing all influenza A subtypes. Cell. (2016) 166:596–608. 10.1016/j.cell.2016.05.073 27453466 PMC4967455

[30] Corti D Voss J Gamblin SJ Codoni G Macagno A Jarrossay D. A neutralizing antibody selected from plasma cells that binds to group 1 and group 2 influenza A hemagglutinins. Science. (2011) 333:850–6. 10.1126/science.1205669 21798894

[31] Richards JO Karki S Lazar GA Chen H Dang W Desjarlais JR. Optimization of antibody binding to FcgammaRIIa enhances macrophage phagocytosis of tumor cells. Mol Cancer Ther. (2008) 7:2517–27. 10.1158/1535-7163.MCT-08-0201 18723496

[32] DiLillo DJ Ravetch JV. Differential Fc-receptor engagement drives an anti-tumor vaccinal effect. Cell. (2015) 161:1035–45. 10.1016/j.cell.2015.04.016 25976835 PMC4441863

[33] Bournazos S Corti D Virgin HW Ravetch JV. Fc-optimized antibodies elicit CD8 immunity to viral respiratory infection. Nature. (2020) 588:485–90. 10.1038/s41586-020-2838-z 33032297 PMC7672690

[34] Micci L Alvarez X Iriele RI Ortiz AM Ryan ES McGary CS. CD4 depletion in SIV-infected macaques results in macrophage and microglia infection with rapid turnover of infected cells. PLoS Pathog. (2014) 10:e1004467. 10.1371/journal.ppat.1004467 25356757 PMC4214815

[35] Metzner KJ Jin X Lee FV Gettie A Bauer DE Di Mascio M. Effects of *in vivo* CD8(+) T cell depletion on virus replication in rhesus macaques immunized with a live, attenuated simian immunodeficiency virus vaccine. J Exp Med. (2000) 191:1921–31. 10.1084/jem.191.11.1921 10839807 PMC2213531

[36] Ortiz AM Klatt NR Li B Yi Y Tabb B Hao XP. Depletion of CD4(+) T cells abrogates post-peak decline of viremia in SIV-infected rhesus macaques. J Clin Invest. (2011) 121:4433–45. 10.1172/JCI46023 22005304 PMC3204830

[37] Schmitz JE Johnson RP McClure HM Manson KH Wyand MS Kuroda MJ. Effect of CD8^+^ lymphocyte depletion on virus containment after simian immunodeficiency virus SIVmac251 challenge of live attenuated SIVmac239delta3-vaccinated rhesus macaques. J Virol. (2005) 79:8131–41. 10.1128/JVI.79.13.8131-8141.2005 15956558 PMC1143721

[38] Pedreno-Lopez N Dang CM Rosen BC Ricciardi MJ Bailey VK Gutman MJ. Induction of transient virus replication facilitates antigen-independent isolation of SIV-specific monoclonal antibodies. Mol Ther Methods Clin Dev. (2020) 16:225–37. 10.1016/j.omtm.2020.01.010 32083148 PMC7021589

[39] Sutton MS Ellis-Connell A Balgeman AJ Barry G Weiler AM Hetzel SJ. CD8beta depletion does not prevent control of viral replication or protection from challenge in macaques chronically infected with a live attenuated simian immunodeficiency virus. J Virol. (2019) 93:e00537-19. 10.1128/JVI.00537-19 31092584 PMC6639280

[40] Martins MA Tully DC Shin YC Gonzalez-Nieto L Weisgrau K Bean DJ. Rare control of SIVmac239 infection in a vaccinated rhesus macaque. AIDS Res Hum Retroviruses. (2017) 33:843–58. 10.1089/AID.2017.004610.1089/AID.2017.0046 28503929 PMC5564033

[41] Huang KH Bonsall D Katzourakis A Thomson EC Fidler SJ Main J. B-cell depletion reveals a role for antibodies in the control of chronic HIV-1 infection. Nat Commun. (2010) 1:102. 10.1038/ncomms1100 20981030 PMC2963804

[42] Gaufin T Pattison M Gautam R Stoulig C Dufour J MacFarland J. Effect of B-cell depletion on viral replication and clinical outcome of simian immunodeficiency virus infection in a natural host. J Virol. (2009) 83:10347–57. 10.1128/JVI.00880-09 19656874 PMC2753117

[43] Tasca S Zhuang K Gettie A Knight H Blanchard J Westmoreland S. Effect of B-cell depletion on coreceptor switching in R5 simian-human immunodeficiency virus infection of rhesus macaques. J Virol. (2011) 85:3086–94. 10.1128/JVI.02150-10 21248033 PMC3067891

[44] Montaudouin C Anson M Hao Y Duncker SV Fernandez T Gaudin E. Quorum sensing contributes to activated IgM-secreting B cell homeostasis. J Immunol. (2013) 190:106–14. 10.4049/jimmunol.1200907 23209322 PMC3572529

[45] Martins MA Shin YC Gonzalez-Nieto L Domingues A Gutman MJ Maxwell HS. Vaccine-induced immune responses against both Gag and Env improve control of simian immunodeficiency virus replication in rectally challenged rhesus macaques. PLoS Pathog. (2017) 13:e1006529. 10.1371/journal.ppat.1006529 28732035 PMC5540612

[46] Moldt B Rakasz EG Schultz N Chan-Hui PY Swiderek K Weisgrau KL. Highly potent HIV-specific antibody neutralization *in vitro* translates into effective protection against mucosal SHIV challenge *in vivo*. Proc Natl Acad Sci USA. (2012) 109:18921–5. 10.1073/pnas.1214785109 23100539 PMC3503218

[47] Mascola JR Lewis MG Stiegler G Harris D VanCott TC Hayes D. Protection of Macaques against pathogenic simian/human immunodeficiency virus 89.6PD by passive transfer of neutralizing antibodies. J Virol. (1999) 73:4009–18. 10.1128/JVI.73.5.4009-4018.1999 10196297 PMC104180

[48] Hessell AJ Rakasz EG Poignard P Hangartner L Landucci G Forthal DN. Broadly neutralizing human anti-HIV antibody 2G12 is effective in protection against mucosal SHIV challenge even at low serum neutralizing titers. PLoS Pathog. (2009) 5:e1000433. 10.1371/journal.ppat.1000433 19436712 PMC2674935

[49] Poignard P Moldt B Maloveste K Campos N Olson WC Rakasz E. Protection against high-dose highly pathogenic mucosal SIV challenge at very low serum neutralizing titers of the antibody-like molecule CD4-IgG2. PLoS ONE. (2012) 7:e42209. 10.1371/journal.pone.0042209 22848744 PMC3407103

[50] Schneider JR Carias AM Bastian AR Cianci GC Kiser PF Veazey RS. Long-term direct visualization of passively transferred fluorophore-conjugated antibodies. J Immunol Methods. (2017) 450:66–72. 10.1016/j.jim.2017.07.009 28780040 PMC5603196

[51] Ackerman ME Moldt B Wyatt RT Dugast AS McAndrew E Tsoukas S. A robust, high-throughput assay to determine the phagocytic activity of clinical antibody samples. J Immunol Methods. (2011) 366:8–19. 10.1016/j.jim.2010.12.016 21192942 PMC3050993

[52] Montefiori DC. Measuring HIV neutralization in a luciferase reporter gene assay. Methods Mol Biol. (2009) 485:395–405. 10.1007/978-1-59745-170-3_26 19020839

[53] Li H Wang S Kong R Ding W Lee FH Parker Z. Envelope residue 375 substitutions in simian-human immunodeficiency viruses enhance CD4 binding and replication in rhesus macaques. Proc Natl Acad Sci USA. (2016) 113:E3413–22. 10.1073/pnas.1606636113 27247400 PMC4914158

