# Supplementary material for: Non-neutralizing Antibodies May Contribute to Suppression of SIVmac239 Viremia in Indian Rhesus Macaques
Source: Front Immunol. 2021 Mar 16;12:657424. doi: 10.3389/fimmu.2021.657424 (PMC8008062; doi:10.3389/fimmu.2021.657424)
Supplement: Supplementary file 1 [file Data_Sheet_1.PDF]

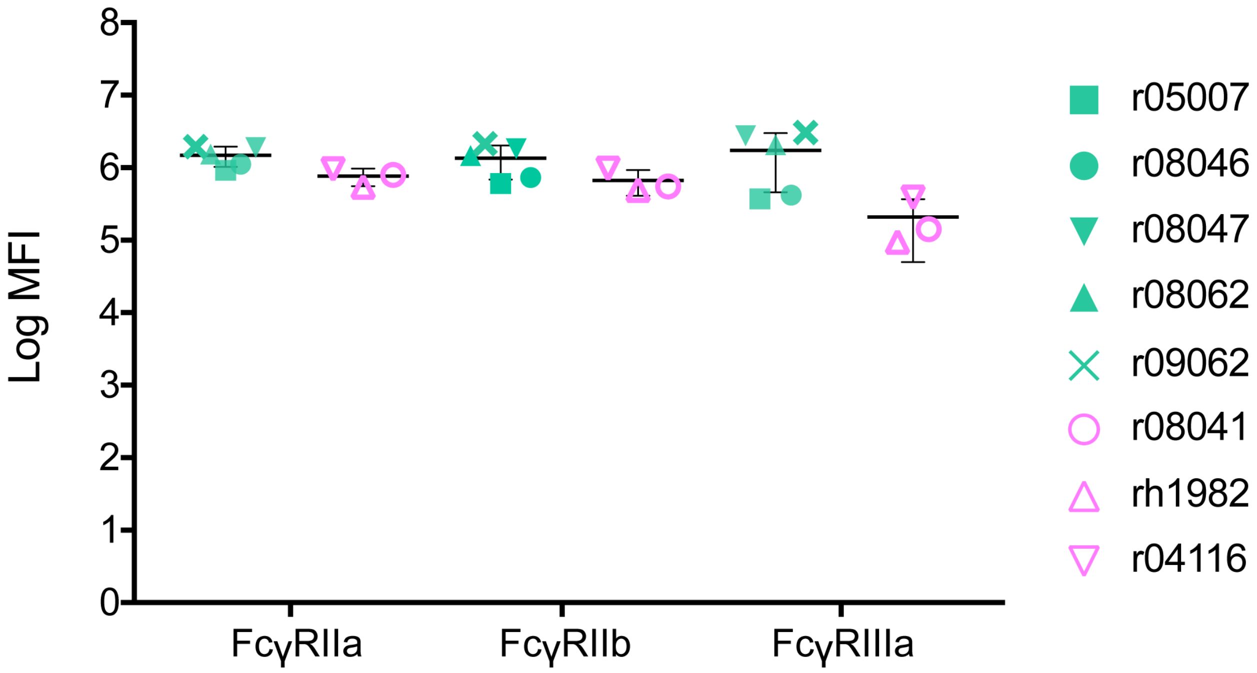

Supplemental Figure 1. Anti-gp140 Abs in the sera of *Mamu-B*17*+ animals bind to human FcγRIIa, FcγRIIb and FcγRIIIa at 1: 500 dilution. Rapid controllers (n = 5) and non-controllers (n = 3) are shown in green and pink, respectively. Mean log MFI for negative controls was 3.7.

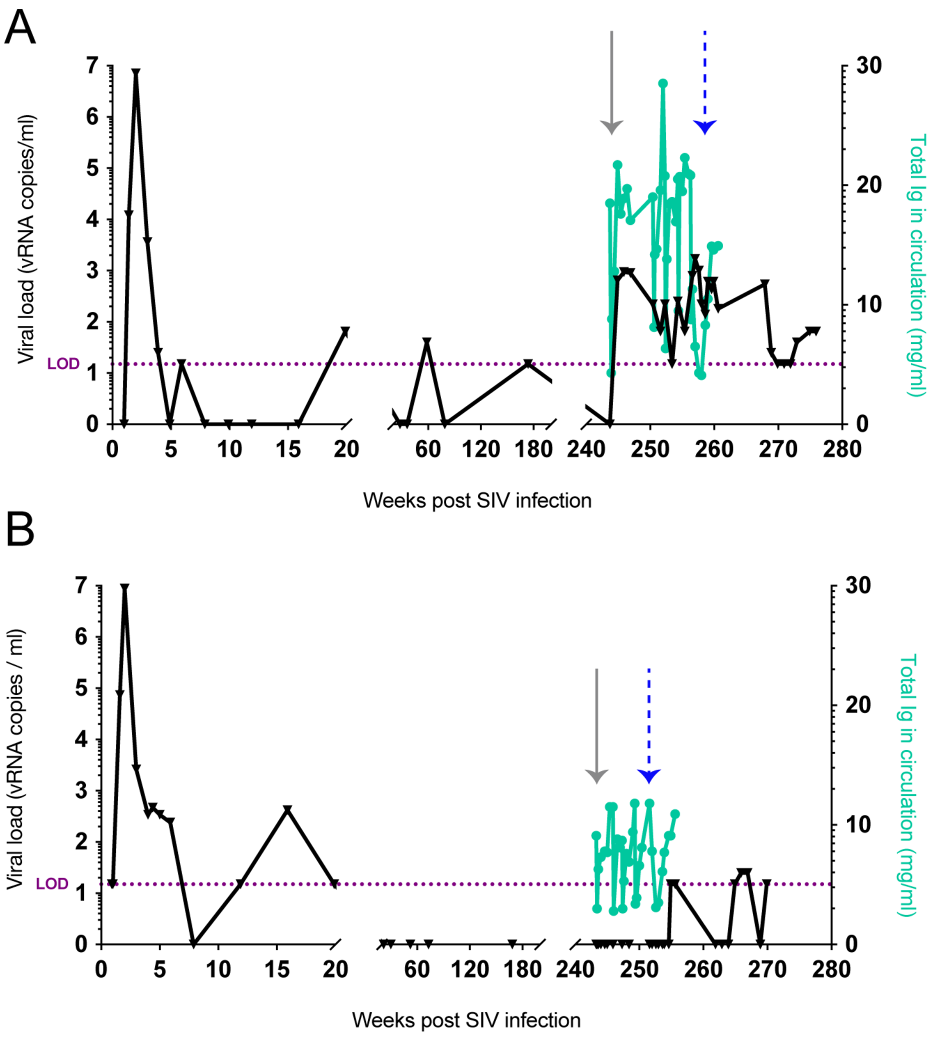

Supplemental Figure 2. IgG levels and viral loads for r05007 and r09062 upon SIV infection. Longitudinal viral loads for A) r05007 and B) r09062 from initial infection through the present study. IgG levels during the immunoadsorption and Ab treatment are indicated in green. Viral loads limit of detection is shown as purple dotted line. Grey arrow indicates the first immunoadsorption procedure. Blue dashed arrow shows the administration of the FcRn Ab.

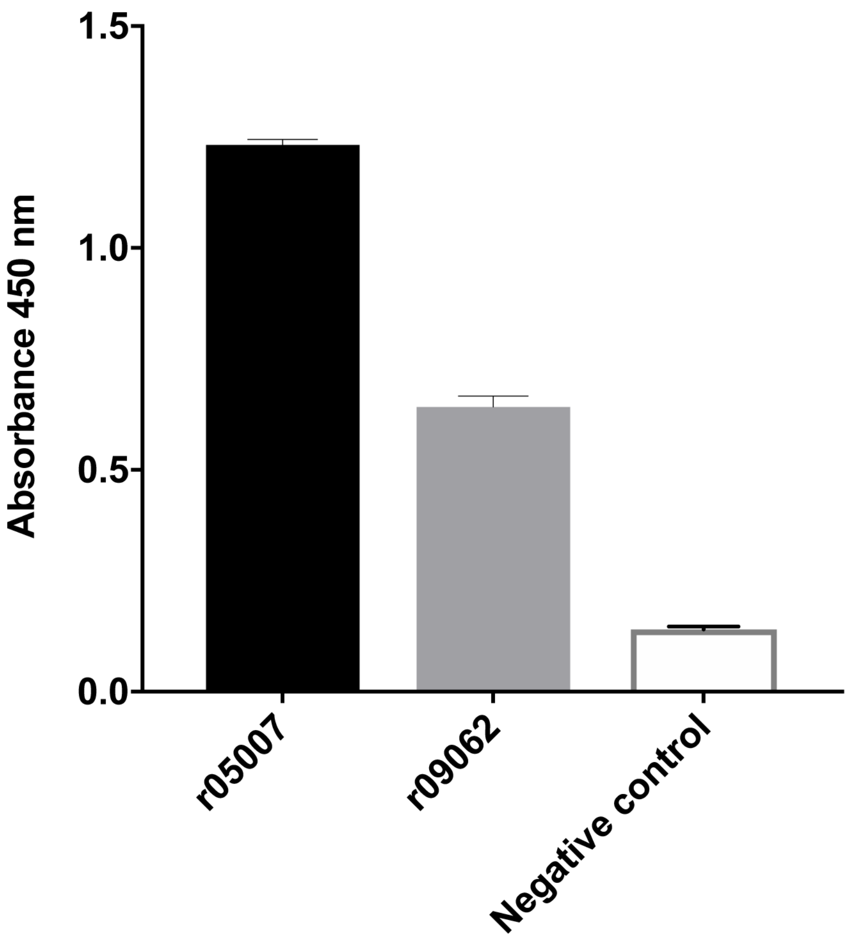

Supplemental Figure 3. Detection of antidrug Abs in RM serum three months following passive infusion of anti-FcRn mAb. RM serum was assayed for antidrug Abs by ELISA. Serum from a SIV-infected RM that did not receive an anti-FcRn mAb infusion was used as a negative control.
